# Psychobiotics and Mental Health in 2024 Among Romanian Consumers: A Cross‐Sectional Survey

**DOI:** 10.1002/hsr2.71399

**Published:** 2026-03-28

**Authors:** Cocean Ana‐Maria, Bernadette‐Emoke Teleky, Dan Cristian Vodnar

**Affiliations:** ^1^ Department of Food Science and Technology, Life Science Institute University of Agricultural Sciences and Veterinary Medicine Cluj‐Napoca Calea Mănăștur Romania

**Keywords:** anxiety, consumer behavior, depression, evidence, probiotics, psychobiotics

## Abstract

**Background and Aims:**

Mental health conditions such as anxiety and depression are prevalent, most notably among vulnerable populations. Psychobiotics are live microorganisms that, when ingested in adequate amounts, may confer mental health benefits via the gut‐brain axis. They represent a promising but poorly understood intervention. This study aimed to assess consumer knowledge about psychobiotics and explore the prevalence of anxiety and depressive symptoms, as well as the influence of socio‐demographic factors on mental health awareness.

**Methods:**

A cross‐sectional online survey was conducted among 427 participants, including Romanian residents (87.1%) and Romanians living abroad (12.9%). The questionnaire gathered information on psychobiotic awareness, mental health knowledge, and self‐reported symptoms of anxiety and depression over the past 6 months. Statistical analyses were performed to identify associations between socio‐demographic factors (age, gender, education, and geographic location) and mental health outcomes.

**Results:**

Although 60% of the respondents were familiar with probiotics, only 18.4% reported being very familiar with psychobiotics. This difference was statistically significant (*p* < 0.001), reflecting a clear disparity in public knowledge between these two terms. Occasional depressive symptoms (26.8%) were more frequently reported than occasional anxiety (23.2%), yet anxiety was more common than depression when experienced regularly (13.8% vs. 10.3%). Notably, 64.2% of respondents stated that reliable information about psychobiotics is lacking in Romania.

**Conclusions:**

These findings show limited public awareness of psychobiotics, despite the growing interest in gut and brain health. Targeted public health and educational initiatives are necessary to close this knowledge gap and promote psychobiotics as a potential tool for enhancing mental health. Integrating information about psychobiotics into broader preventive health strategies could improve mental health literacy, foster informed choices, and support early interventions for anxiety and depression.

## 1 Introduction

**Figure 1:**
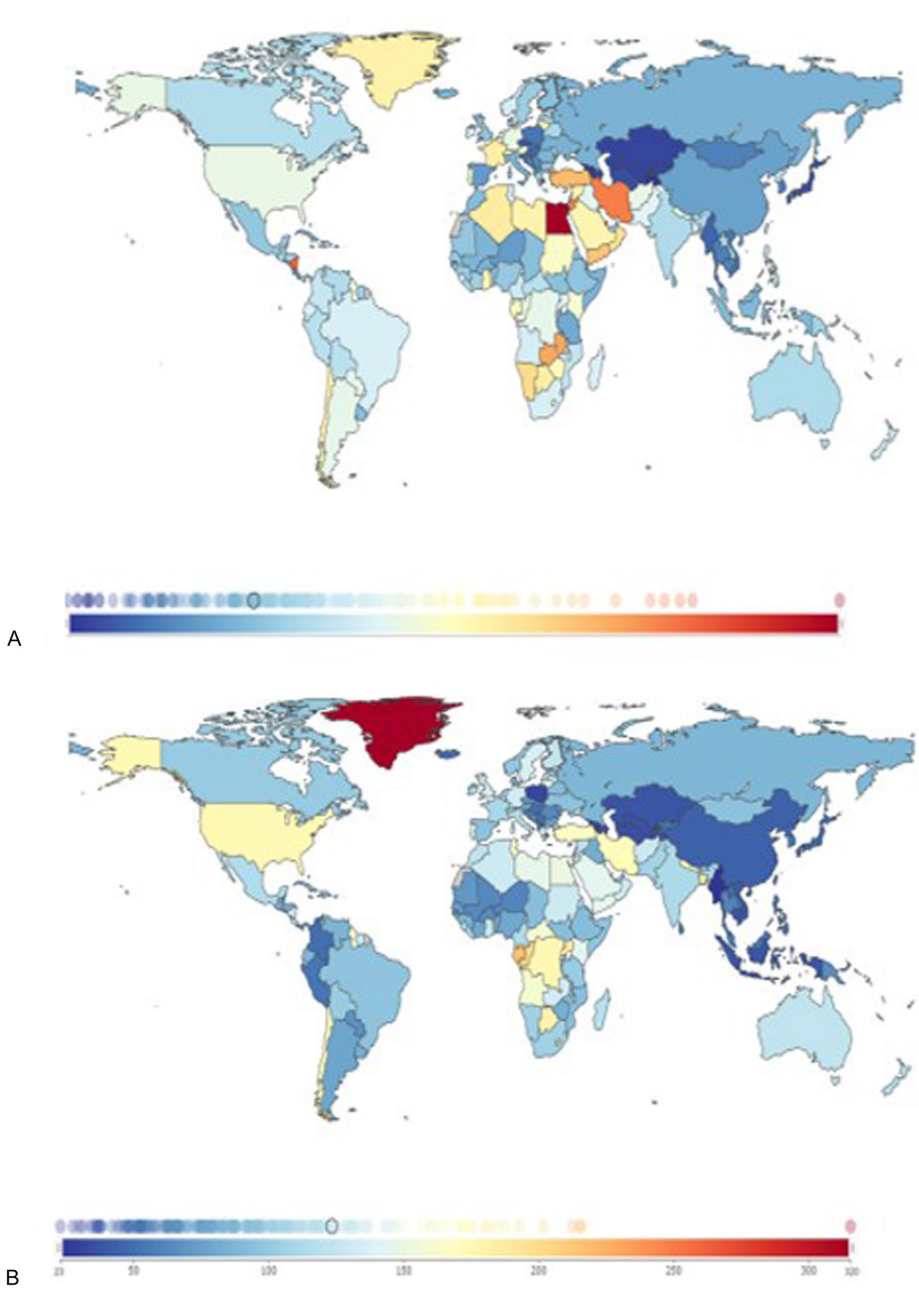
Global incidence of mental health disorders attributable to all risk factors in 2021. (A) Anxiety disorders and (B) depressive disorders, expressed as Years Lived with Disability (YLDs). Color gradients represent burden intensity, with lighter shades indicating lower YLDs and darker/redder shades indicating higher YLDs. Data includes both sexes and all age groups. Source: Institute for Health Metrics and Evaluation (IHME), Global Burden of Disease (GBD) Compare Tool (https://vizhub.healthdata.org/gbd-compare).

Romania's accession to the European Union in 2007 has aligned it with broader European health standards; however, mental health issues remain a pressing concern [1]. Globally, mental health conditions such as anxiety and depression have worsened in recent years, with the COVID‐19 pandemic further accelerating this trend. According to the World Health Organization (WHO), the demand for mental health care continues to rise. At the same time, resources and support systems remain insufficient to meet the growing need [2]. Currently, an estimated 970 million people worldwide are affected by mental disorders, with depression and anxiety being the most prevalent [3]. This growing burden underscores the urgency of developing cost‐effective, scalable, and accessible strategies for the prevention and management of common mental disorders. Data from the Global Burden of Disease (GBD) Compare tool (Figure 1) show a significant post‐pandemic increase in anxiety disorders, with notable regional differences in incidence. Similar increasing trends are observed for depressive disorders, which affect all age groups, but are particularly prevalent among young people and women.

Depression and anxiety are intricate and influenced by multiple factors. Various factors, including diet, stress, medication, genetics, and the gut microbiota, contribute to the occurrence or absence of symptoms [4]. Mood disorders, including sadness and anhedonia, as well as cognitive deficits, impaired performance, and low self‐esteem, are common signs of depression. Depression is also a significant contributor to suicide attempts [5]. On the other hand, generalized anxiety disorder (GAD) is a prevalent chronic mental illness characterized by symptoms of physiological arousal, such as anxiety, insomnia, and muscle tension, as well as symptoms of excessive worry. Furthermore, anxiety disorders rank sixth among all causes of impairment in the Global Burden of Disease study. The prevalence of these disorders is highest among women and those aged 15–34 years [6].

Recent research into the gut–brain axis has revealed a profound connection between the gut microbiota and mental health, facilitated by a dynamic two‐way communication system linking the gut to the brain. Studies have highlighted the impact of gut bacteria on brain function and connectivity, potentially contributing to various aspects of brain development, cognition, and neurological disorders [7, 8]. Through the production of bioactive substances, the gut microbiota can modulate cells in the body, communicate via the bloodstream and nerves, and interact with immune system cells, ultimately shaping emotions, cognitive processes, and susceptibility to psychiatric conditions [8, 9].

In recent years, there has been growing interest in the role of the gut microbiota in human health, particularly in its influence on brain function through the gut–brain axis. This bidirectional communication system connects the gastrointestinal tract and the central nervous system via neurological, endocrine, immune, and metabolic pathways [8, 10, 11, 12]. While the precise mechanisms remain under investigation, evidence increasingly suggests that gut bacteria can influence mental well‐being.

The World Health Organization defines probiotics as live microorganisms that confer health benefits to the host when administered in adequate amounts. The term “probiotic,” derived from the Greek meaning “for life,” highlights their beneficial role in promoting overall health [13]. In 2013, the concept of psychobiotics was introduced to describe a subclass of probiotics capable of producing neuroactive compounds that positively influence mood, cognition, and stress‐related behavior [14]. Foundational work by Cryan and colleagues had already laid the scientific groundwork for this field, demonstrating how gut microorganisms influence brain development, stress regulation, and emotional behavior, thus solidifying the gut–brain axis as a target in neuropsychiatric research [15, 16]. These microorganisms may modulate the hypothalamic–pituitary–adrenal (HPA) axis, attenuating the stress response and reducing inflammation associated with its dysregulation [17, 18]

Multiple mechanisms have been proposed to explain psychobiotic action. One involves the modulation of the hypothalamic‐pituitary‐adrenal (HPA) axis, which plays a crucial role in stress regulation. Psychobiotics may attenuate the stress response and reduce inflammation associated with the dysregulation of this axis [19, 20]. Another pathway relates to the immune system, as psychobiotics can modulate cytokine production and inflammation, processes known to influence mood and emotional health [21]. Additionally, these microorganisms secrete bioactive compounds such as neurotransmitters (e.g., GABA, serotonin), proteins, and short‐chain fatty acids (SCFAs), which may affect brain function after being absorbed into systemic circulation and transported to the brain, where they interact with components of the central nervous system [22].

The relationship between the gut microbiota, the HPA axis, and cognitive processes is bidirectional and mediated through a complex network of interconnected pathways. These pathways encompass the vagus nerve, neurotransmitter and metabolite synthesis, immune system regulation, blood–brain barrier function, and hormone metabolism. By targeting these pathways, psychobiotics may exert their beneficial effects on mental health [13].

**Figure 2:**
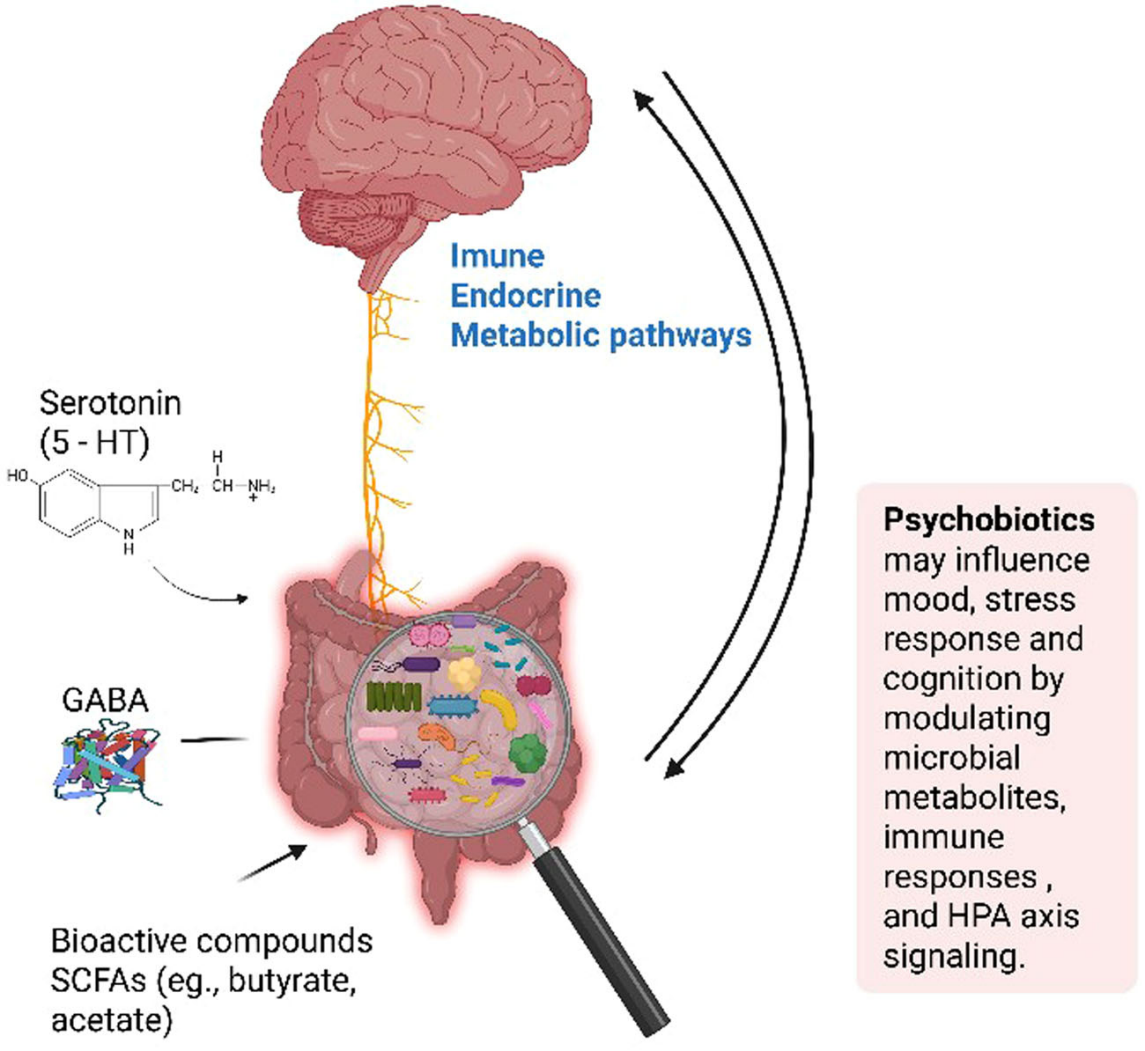
Bidirectional communication between the gut microbiota and the brain.

This bidirectional communication between the gut and the brain is summarized in Figure 2, which highlights the primary psychobiotic mechanisms, including immune and metabolic signaling, as well as the production of neurotransmitters such as serotonin and gamma‐aminobutyric acid (GABA).

To the best of our knowledge, there have been no studies specifically focused on awareness of psychobiotics. However, multiple studies have been conducted in various countries worldwide to examine the awareness and consumption of probiotics and prebiotics. For example, a survey conducted in the United Arab Emirates in 2024 showed that 33.7% of participants possessed substantial knowledge of probiotics [23]. Similarly, a 2021 survey of pediatricians in Saudi Arabia found that 57.7% of 452 respondents knew the definition of probiotics, understanding them as live microorganisms that confer a health benefit to the host [24] when administered in sufficient quantities. Additionally, a study was conducted in Romania to assess consumer awareness, understanding, and interest in prebiotics. The results showed that participants' levels of knowledge were contradictory. Age and knowledge levels were significantly correlated, suggesting that younger participants had a better understanding and were more aware of prebiotics than older participants (*p* < 0.05) [25].

Despite the availability of psychobiotics in Romanian pharmacies and online marketplaces, there needs to be more public awareness and information about these products. Addressing these knowledge gaps is crucial for developing educational materials that clearly define, highlight the benefits, and illustrate the applications of psychobiotics. This study aims to assess Romanian consumers' perceptions, knowledge, and potential barriers regarding psychobiotics, to improve public awareness and foster informed decision‐making in mental health care.

## 2 Materials and Methods

### 2.1 Participants

This cross‐sectional study aimed to examine Romanian consumers' understanding, use, and perception of psychobiotics. Participants, including Romanians living abroad and in Romania, were asked to complete an anonymous online survey. Both urban and rural areas were represented among the participants. The survey instrument was distributed through email and social media. Data was collected from May 2024 to July 2024. Participants were divided into two groups: young consumers (aged 15–24) and adults (aged 25–64). Before beginning the questionnaire, all participants were presented with an informed consent statement. By voluntarily completing the survey, they confirmed their consent to participate, and their responses were collected anonymously without any identifying information.

### 2.2 Study Design

The study employed a comprehensive, multi‐part questionnaire as the survey instrument, comprising 26 questions. These included closed‐ended questions (with predetermined response options) and open‐ended questions (allowing respondents to provide detailed answers in their own words). This varied range of question types aimed to collect quantitative and qualitative data for an in‐depth understanding of the topic. This study adheres to the STROBE guidelines for cross‐sectional studies to enhance the clarity, transparency, and reproducibility of its findings [26].

The estimated time to complete the questionnaire was 10–12 min. The answers were accurate and easy to understand as all participants used the Romanian version of the questionnaire (https://forms.gle/e9ENSEChzqfWMtTS6), eliminating any possible language barrier. The online questionnaire began with a consent form that all participants were required to fill out. To ensure response accuracy, the survey incorporated screening questions and logical validations to prevent the submission of contradictory answers. Incomplete or inconsistent responses were also excluded from the analyzed data.

The first section of the survey collected basic demographic details, including age, gender, education, and place of residence. The second part assessed participants' knowledge of probiotics and psychobiotics, familiarity with these terms, and awareness of associated health benefits. Also included in the survey were participants' expectations of psychobiotic products, their views on the availability of information about psychobiotics in Romania, and their overall perception of psychobiotics.

Following this, the survey sought respondents' psychological well‐being by asking them about their thoughts and feelings about the effectiveness of psychobiotics in treating mental health problems, including depression and anxiety. In addition, the questionnaire examined factors that may influence consumers' decisions to purchase psychobiotic products, such as price, professional recommendations, and the presence of scientific evidence supporting the efficacy of these products. Participants were also asked to identify the sources of information they considered most reliable for their information about the benefits of psychobiotic products. The survey concluded by encouraging participants to express their general impressions, including their preferred methods of consuming psychobiotics and any other comments or observations they wished to share.

### 2.3 The Sample Size Estimation

A well‐established formula used in survey research was used to determine the appropriate sample size for estimating the population mean [27]. The sample size calculation was based on Romania's total adult population (*N*), estimated to be 15 million people. Equation (1) was used to calculate the sample size appropriate for estimating the population mean in our study:

(1) $$N=\frac{Z^{2}*p*(1-p)}{E^{2}}$$

In the above formula: *n* is the sample size (number of respondents needed for the study) *Z* is the *Z*‐score indicating the desired confidence level (e.g., 1.96 for a 95% confidence level), *p* is the estimated proportion of population prevalence of the topic of interest, and *E* is the margin of error (the maximum accepted difference between the sample size and the proper population proportion).

Rounded to a whole number, the sample size needed to obtain nationally representative results, with a 95% confidence level and a 5% margin of error, is approximately 384 respondents. This sample size will ensure that the data collected through the questionnaire will be sufficiently accurate and generalizable to the adult population in Romania.

### 2.4 Statistical Analysis

The demographics of the survey participants were summarized using descriptive statistics. Continuous variables, such as age and Likert‐scale responses (e.g., familiarity with psychobiotics, confidence in their mental health benefits), were summarized using either the mean and standard deviation (SD) for normally distributed data or the median and interquartile range (IQR) for non‐normally distributed data, as determined by normality testing. Categorical variables (e.g., gender, education level, geographic location, awareness of psychobiotics) were summarized using frequency counts and percentages.

The primary outcomes of the survey included assessing the associations between socio‐demographic characteristics (age, gender, education level, and geographic location) and respondent factors such as psychobiotic awareness, frequency of anxiety/depression symptoms, and help‐seeking behavior. Group comparisons were performed using one‐way analysis of variance (ANOVA), followed by Tukey's post hoc test, using GraphPad Prism Version 9.3.0 (GraphPad Software Inc., San Diego, CA, USA), with statistical significance defined as *p* < 0.05. Confidence intervals (CIs) were provided where appropriate to indicate the precision of estimates.

## 3 Results and Discussions

### 3.1 Demographic Profile of Respondents

The demographic background of the study's participants is summarized in Table 1. The online survey yielded 427 completed questions. A total of 427 respondents fully completed all sections of the questionnaire. The sample consisted of 74.0% (*n* = 316/427) females and 26.0% (*n* = 111/427) males. The median age of participants was 30 years (IQR: 22–38). The respondents were divided into two groups: young consumers (aged 15–25) and adults (aged 26–64). Young consumers comprised 38.4% (*n* = 164/427) of the respondents, with an average age of 22 years, while adults comprised 61.6% (*n* = 263/427), with an average age of 38 years. Male participants accounted for 26% (*n* = 111/427) of the total, while female participants constituted 74% (*n* = 316/427). Similarly to previous research, most participants in our sample were females [19, 20]. Furthermore, 87.1% (*n* = 372/427) of the respondents lived in Romania, while Romanians residing in other countries comprised 12.9% (*n* = 55/427) of the total, with the majority living in Spain, Germany, Italy, the United Kingdom, Ireland, the Netherlands, and Cyprus.

**Table 1:** Study participant's demographic information.

Characteristic	*n*	%
Age group		
Young individuals (15–25 years old)	164	38.4
Adults (26–64 years old)	263	61.6
Gender		
Male	111	26
Female	316	74
Geographical location		
Rural	157	36.9
Urban	270	63.1
Countries		
Romania	370	87.1
Abroad		
Italy	11	2.58
Spain	4	0.94
UK	3	0.70
Ireland	2	0.47
Germany	10	2.35
Netherlands	3	0.70
Cyprus	1	0.23
Other countries	22	5.16
Level of qualification		
University studies	319	74.7
Postsecondary education	10	2.34
High school	86	20.14
Vocational school	12	2.81

The survey results indicated that most respondents, representing 74.9% (*n* = 320/427) of the participants, had higher education qualifications, including bachelor's, master's, and/or doctoral degrees, making this group the largest within the sample. The second largest group, comprising 20% (*n* = 85/427) of the participants, had completed secondary education at the high school level. A small minority, 2.6% (*n* = 11/427), reported having only secondary education, while only 2% (*n* = 9/427) of respondents had completed primary education as their highest level of schooling.

In terms of gender, the sample was predominantly female, with women accounting for 74% of participants. This notable imbalance may introduce sampling bias, particularly given the well‐established gender differences in health‐related behaviors. Research indicates that women are more likely to seek health information and engage with mental health topics than men, which may have influenced the levels of awareness, interest, and engagement reported in this study. Consequently, the findings may not be fully generalizable to male and gender‐diverse populations [28].

Research evidence consistently shows that higher levels of education have positive long‐term effects on mental health. For example, an additional year of tertiary education is associated with a significantly reduced risk and severity of depression and anxiety. These effects are particularly pronounced for women and rural populations, likely due to better physical health, healthier lifestyle choices, and greater personal autonomy [29, 30].

WHO states that everyone has the inherent right to be healthy, defined as “a state of complete physical, mental, and social well‐being and not merely the absence of disease or infirmity” [31]. This definition highlights the importance of considering health as a multifaceted concept that extends beyond physical conditions.

### 3.2 Knowledge of Probiotics and Psychobiotics

**Figure 3:**
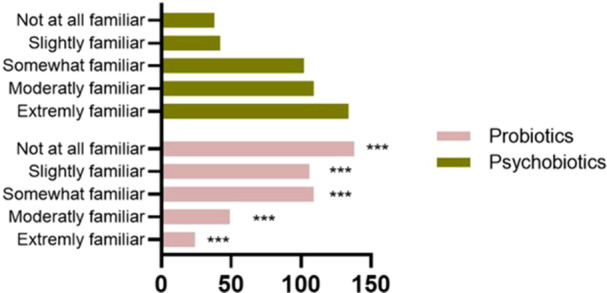
The knowledge level of the terms “Probiotics” and “Psychobiotics”. Tukey's multiple comparison tests were applied, with *p* values provided when *p* > 0.05. The following symbol was used for interpretation: ***p < 0.001.

When respondents were asked to rate their familiarity with probiotics on a scale of 1–5 (1—Not at all familiar; 2—Somewhat familiar; 3—Moderately familiar; 4—Very familiar; 5—Extremely familiar), 32.4% (*n*  =  138/427) of respondents said they were extremely familiar, while almost 5.6% (*n* = 24/427) said they were not familiar at all (Figure 3). In contrast, when asked about psychobiotics, only 8.9% (*n* = 38/427) of the respondents stated they were highly familiar with this term, while 31.5% (*n* = 134/427) had never heard of it.

Public awareness of the microbiota–gut–brain axis and psychobiotics remains low, not only in Romania but also globally. Survey data and literature reviews consistently indicate that the general population in both developed and developing countries has a limited understanding of how the gut microbiota influences mental health outcomes. Moreover, a bibliometric analysis by Abdill et al. demonstrated that publicly available human microbiome dataare overwhelmingly generated in highly developed countries, resulting in globaldisparities in scientific representation and research visibility [32]. Supporting this observation, the researchers found in their study on maternal health in India that knowledge, attitudes, and practices regarding psychobiotics remain significantly underdeveloped [33].

**Figure 4:**
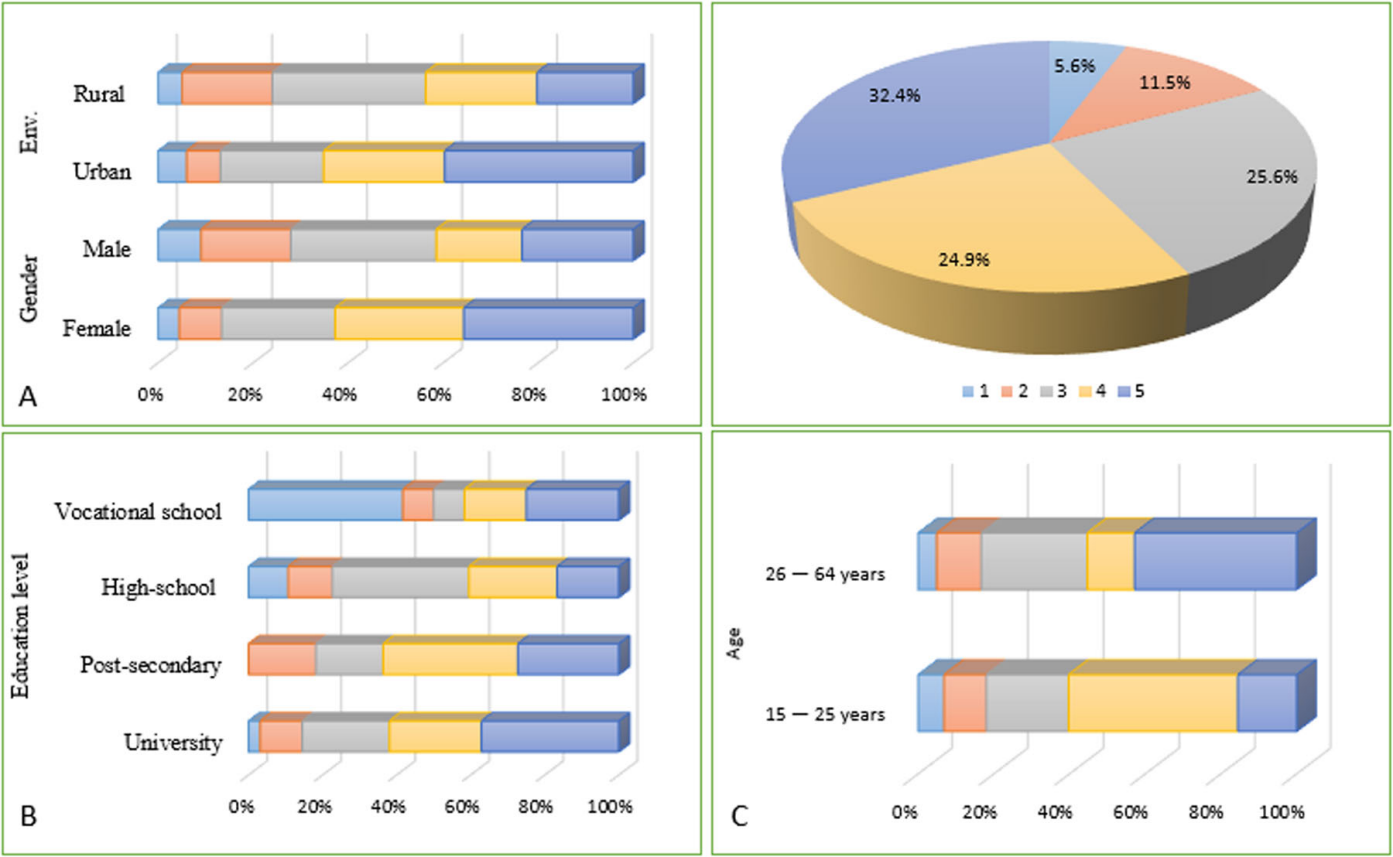
Respondents' degree of familiarity with probiotics, categorized by sociodemographic characteristics. (A) Gender and geographical location (rural vs. urban), (B) educational level, and (C) age group (15–25 years and 26–64 years). Familiarity was rated on a 5‐point Likert scale, where 1 = “to a minimal extent” and 5 = “to a considerable extent.

As illustrated in Figure 4A–C respondents from Romania were categorized based on gender, age, and education level. The distribution of respondents indicates that most participants are significantly more familiar with the term “probiotic” than “psychobiotic” (CI: 6.965–7.035, *p* < 0.001). Across the sample, 60% (*n* = 256/427) of participants reported familiarity levels of 4 or 5 for probiotics, indicating that respondents felt either “very familiar” (level 4) or “extremely familiar” (level 5), which reflects strong prior knowledge and a confident understanding. In contrast, only 18.4% (*n* = 79/427) of participants reached this level of familiarity with psychobiotics, although over 57% (*n* = 244/427) selected levels 1 or 2, indicating minimal or limited awareness. This highlights a substantial gap in public knowledge regarding psychobiotics.

The investigation of familiarity with probiotics, illustrated in Figure 4, reveals significant variations by gender and geographical location. Women are more familiar with probiotics than men (CI: 3.613–117.2, *p* < 0.01). A substantial proportion of female respondents fall into the categories of high familiarity. In contrast, male respondents exhibit a more even distribution across the familiarity categories, with a notable focus on the moderate familiarity category. However, it is essential to note that many men also report high familiarity, indicating that although overall familiarity may be lower among men, a sizable segment possesses substantial knowledge about probiotics.

Furthermore, geographical location plays a vital role in forming familiarity with probiotics. Urban respondents show significantly higher levels of familiarity patterns compared to rural respondents. Within urban populations, the majority fall into the high familiarity categories, reflecting a solid understanding of probiotics. In contrast, rural respondents tend to exhibit lower levels of familiarity, with a higher proportion falling into the lower categories. This heterogeneity may be attributable to differences in access to information and educational resources on probiotics between urban and rural areas.

Current research supports that women are more interested in health information than men. When it comes to taking care of their own and their family's health, studies show that women are more likely to seek out information on this topic and are more engaged in the process in general [34]. Findings from our study confirm this pattern, showing that health issues are of more interest to women than men (CI: 17.11–105.7, *p* < 0.01).

Additionally, a 2024 study highlights the growing popularity of probiotic products in the Hong Kong market, where consumer acceptance is particularly high. Gender, level of education, and income are key factors that significantly influence the use of probiotics and knowledge about them. A relevant demographic that should be targeted is more educated women, due to their greater propensity to understand and adopt these products. However, in the case of this study, many participants still do not know what probiotics are or how to use them properly, despite their widespread use today [35].

**Figure 5:**
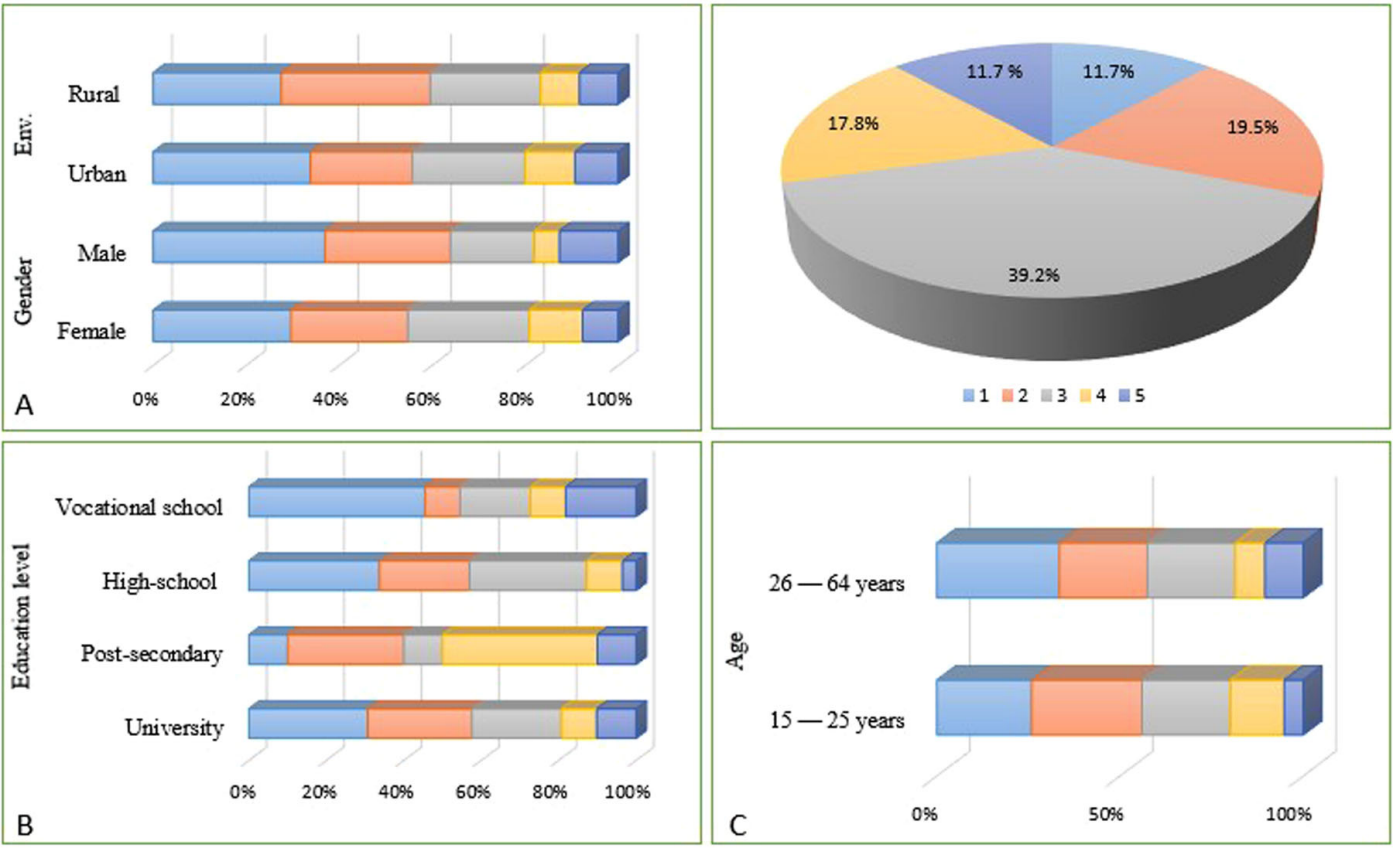
Respondents' degree of familiarity with psychobiotics, categorized by sociodemographic characteristics. (A) Gender and geographical location (rural vs. urban), (B) educational level, and (C) age group (15–25 years and 26–64 years). Familiarity was rated on a 5‐point Likert scale, where 1 = “to a minimal extent” and 5 = “to a considerable extent”.

As shown in Figure 5, a pattern similar to the one observed for probiotics emerges in relation to psychobiotics: women generally report higher familiarity levels than men (CI: 2.509–91.09, *p* < 0.01). This difference reflects a broader trend observed in health research, where women are typically more proactive in seeking and engaging with health‐related information. Rural participants, by contrast, tend to show lower familiarity with psychobiotics, with a significant portion clustered in the lower familiarity categories, reflecting reduced access to health‐related information in these areas.

Figure 5B further illustrates the role of education level in shaping knowledge. Respondents with a university education demonstrated the highest levels of familiarity with psychobiotics (*p* < 0.001), highlighting the strong influence of advanced education on health knowledge. Those with postsecondary (but not university) education demonstrated moderate familiarity, indicating an increasing but incomplete understanding. Respondents with only a high school education reported the lowest levels of familiarity, with the majority of responses falling into the lower categories. Interestingly, vocational school respondents showed a similar pattern to the postsecondary group, with familiarity scores also clustered in the middle range.

Our research shows that awareness of psychobiotics differs considerably in gender, geographic location, educational level, and age (CI: −22.49 to 66.09, *p *< 0.99). As illustrated in Figure 5C, age plays a key role, with individuals aged 26–64 demonstrating a more robust understanding of psychobiotics than younger respondents. This trend suggests that older adults may have greater exposure to health‐related information on psychobiotics, possibly due to heightened health concerns or better access to relevant resources that they have accumulated over time.

**Figure 6:**
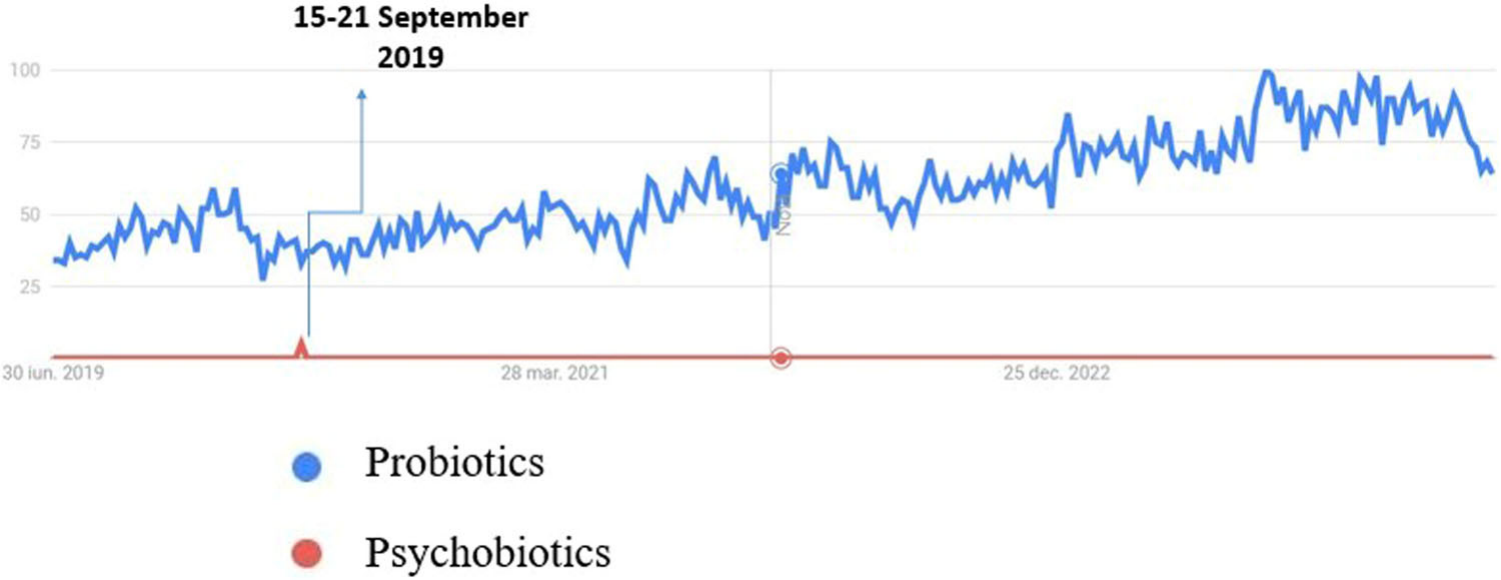
Google Trends comparison of public interest in the terms “probiotics” and “psychobiotics” between June 2019 and July 2024. Data are normalized on a scale from 0 to 100, where 100 indicates peak popularity of the search term during the specified time period. Data source: https://trends.google.com, accessed on July 10, 2024.

Khalesi et al. also point out the general need for gut health knowledge and knowledge about the gut microbiota, prebiotics, and probiotics in the Australian population. Their research found that knowledge of probiotics, prebiotics, natural sources, and guidelines for fruit consumption was positively associated with the use of probiotics. This indicates that people who maintain a healthier lifestyle and better understand gut health are more likely to use probiotics [36]. Moreover, Google Trends data over the past 5 years (Figure 6) indicates that probiotics have consistently garnered more interest than psychobiotics. This imbalance in popularity is also reflected in the scientific literature, where probiotics remain a widely researched and discussed topic.

On the other hand, the term “psychobiotics” has garnered significantly less attention. Psychobiotics, which refer to specific strains of probiotics that have potential mental health benefits, are a relatively new and emerging field. *Lactobacillus* and *Bifidobacteria*'s widely recognized probiotic strains are key in maintaining intestinal and mental health. This is mainly due to their external structure, which lacks the pro‐inflammatory lipopolysaccharide (LPS) chains found in pathogenic bacteria such as *Salmonella* or *Escherichia coli* [37]. Research has identified these strains as psychobiotics due to their potential psychiatric effects on individuals experiencing anxiety and depression [38, 40]. The low interest in psychobiotics will likely reflect limited public awareness and understanding. The new concept is still gaining ground among researchers and health professionals and has not yet reached the same level of general recognition as probiotics. The key point is that interest in probiotics has been consistently high during this period, whereas interest in psychobiotics was only sporadic, particularly in September 2019. This difference can be explained by the fact that probiotics are a more well‐known and publicly discussed concept, while psychobiotics represent a newer and less widespread domain.

**Figure 7:**
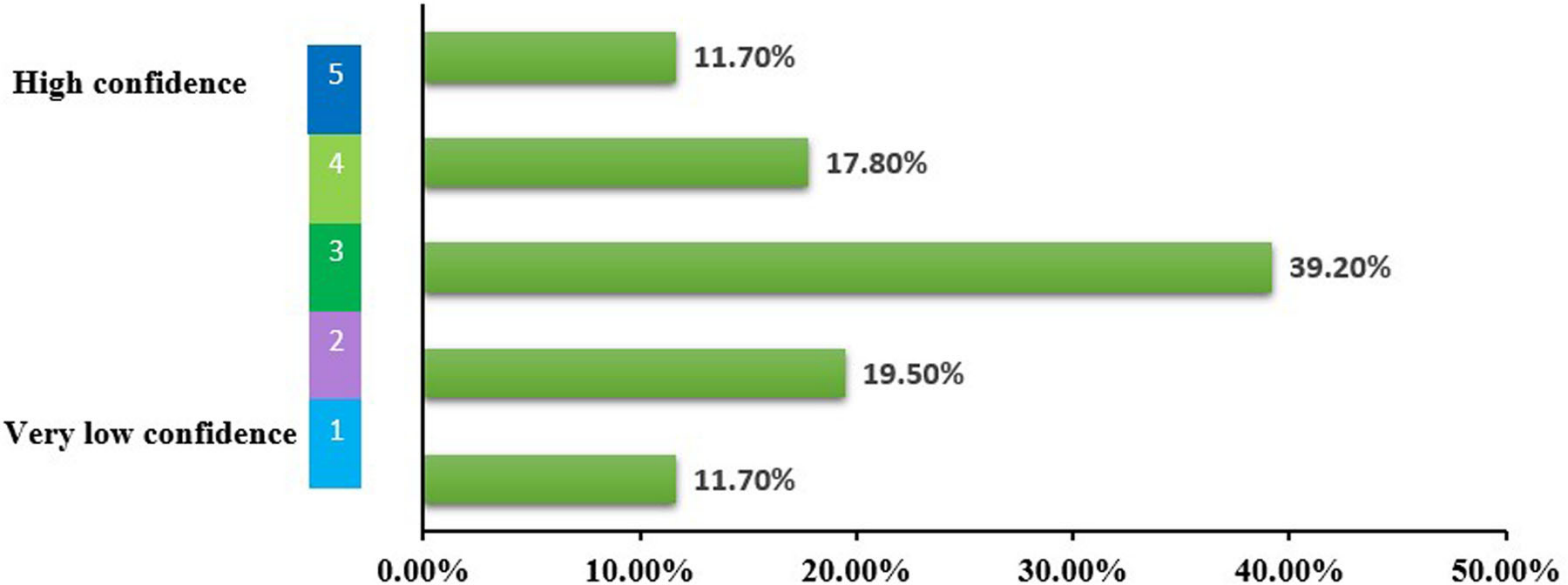
Level of confidence in the mental health benefits of psychobiotics. Data were collected using a 5‐point Likert scale, where 1 = “very low confidence” and 5 = “high confidence.” The figure illustrates the distribution of responses among participants (*n* = 427).

As seen in Figure 7, 12.10% (*n* = 52/427) have no confidence at all in the mental health benefits of psychobiotics, while approximately 14% (*n* = 60/427) have very high confidence. The results indicate a relatively well‐balanced distribution of confidence levels in the mental health benefits of psychobiotics. This distribution suggests that most respondents are moderately confident in the benefits of psychobiotics, with a significant minority expressing lower confidence.

Interestingly, a study conducted primarily among students at the University of Delhi, India, highlights a significant knowledge gap regarding probiotics. It found that 74.38% of students were unaware of the brand names of probiotic supplements, and 72% did not know the recommended dosage or units of measurement for probiotic bacteria. This suggests a lower confidence level in the effective use of probiotics in practice [41]. Most respondents (49.75%) associated probiotics with enhancing immunity and digestive health, indicating confidence in their benefits. However, some respondents were still unaware of these benefits.

Similarly, a study conducted at the University of Rajshahi in Bangladesh, within the Department of Genetic Engineering and Biotechnology, found that, although the participants were students in a biology‐related field, 31.5% were unfamiliar with the term probiotics. This finding is interesting, given their academic background, suggesting a widespread knowledge deficit even among those who should have a fundamental understanding of the topic. However, the study also noted a prominent level of willingness (90.3%) among participants to consume probiotics if advised by a health professional, indicating a reliance on external guidance rather than their knowledge. These collective findings indicate significant gaps in probiotic education and awareness, even among students in related fields, and raise critical questions about the effectiveness of current educational strategies in addressing these gaps [42].

**Figure 8:**
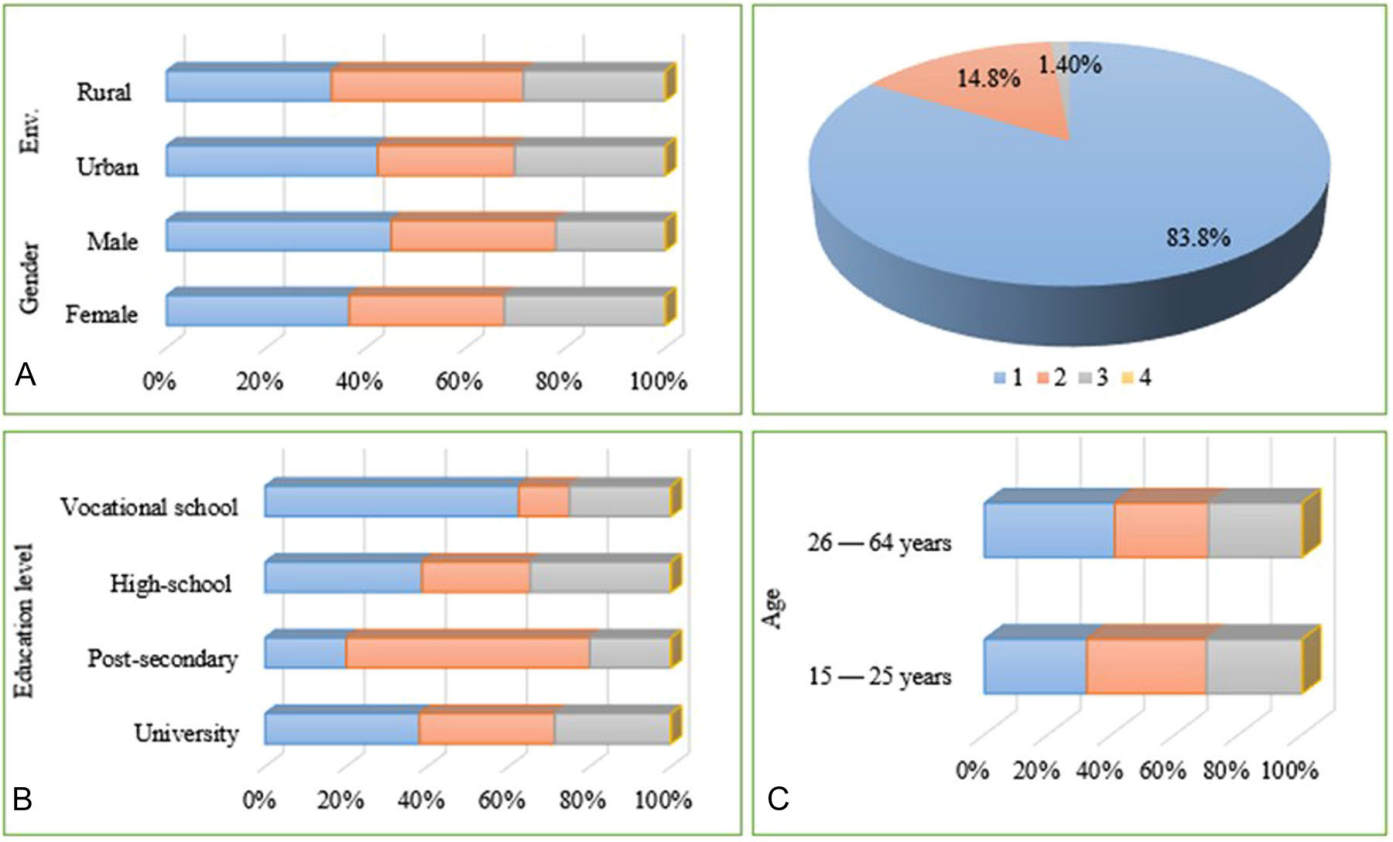
Importance assigned to scientific evidence in proving the efficacy of psychobiotics, based on respondents' perceptions. Responses were measured using a 4‐point Likert scale, where 1 = “very important,” 2 = “moderately important,” 3 = “low importance,” and 4 = “not important at all.” The pie chart displays the overall distribution of perceived importance across the entire sample (*n* = 427). (A) Distribution of responses according to gender and living environment. (B) Distribution of responses according to education level. (C) Distribution of responses according to age group.

We also investigated the importance of scientific evidence demonstrating the efficacy of psychobiotics. As shown in Figure 8, the results are categorized using a 4‐point scale, where 1 indicates “very important,” 2 indicates “moderately important,” 3 indicates “low importance,” and 4 indicates “not important at all.” The data reveal significant differences across demographic groups, as noted. In terms of gender (CI: 4.267–63.07, *p* < 0.01), compared to men, women seem to have a stronger tendency to consider scientific validation particularly important. The patterns indicate that more women than men fall into the higher importance categories. Additionally, people residing in urban areas tend to place a higher value on scientific evidence, whereas those in rural areas are more likely to assign a lower value to it (CI: −11.07 to 47.73, *p* < 0.99). This suggests a possible relationship between urban living and greater exposure to health or science‐related material, which could impact their beliefs about the importance of evidence.

Figure 8B indicates very clearly that the perceived relevance of scientific evidence is positively correlated with higher levels of education. Respondents fill the essential category with a bachelor's degree or more. Still, the distribution is more varied among those with a high school diploma or vocational training, and fewer of these respondents place high importance on scientific validation. This correlation demonstrates the importance of education in shaping perspectives toward scientific facts. There is also a correlation between age and the importance given to scientific evidence, as seen in Figure 8C. People aged 26–64 are more likely to place a high value on evidence, but those aged 15–25 tend to be more balanced. Whether this is due to greater awareness of health issues or wisdom gained from years of experience, this indicates the possibility that people's understanding of and reliance on science‐supported health evidence may increase with age.

### 3.3 Available Information on Psychobiotics

**Figure 9:**
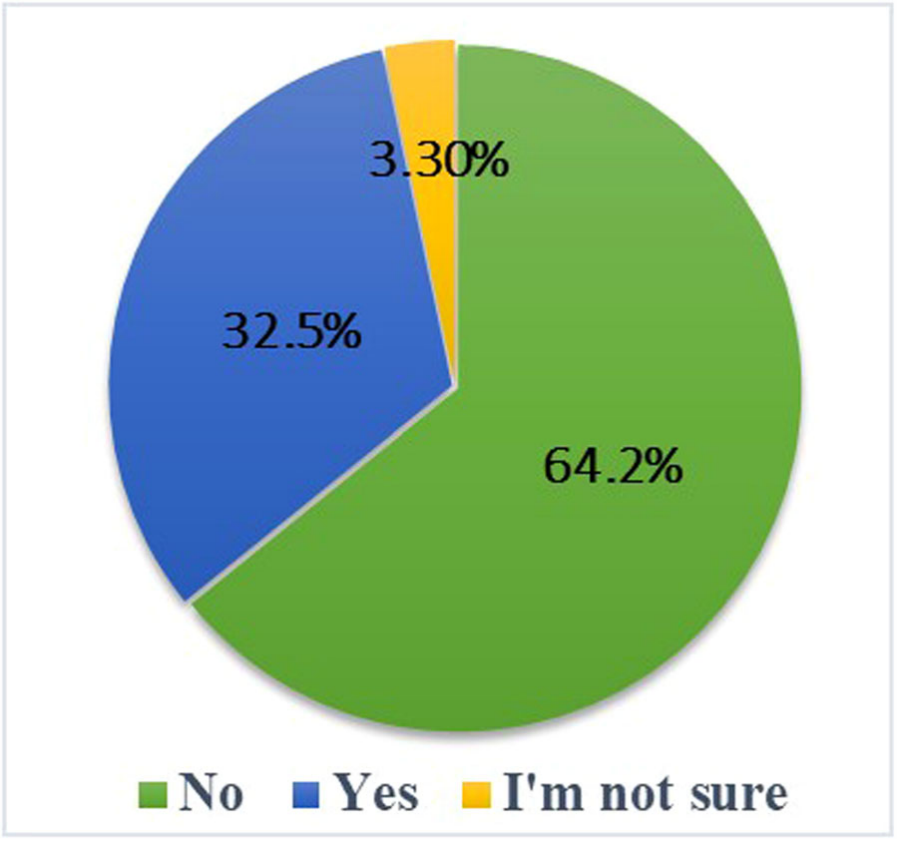
Perceived availability of reliable information on psychobiotics among Romanian respondents based on a sample of 427 participants.

The survey results, presented in Figure 9, indicate that 32.5% (*n*  =  139/427) of respondents in Romania identified a gap in the available information on psychobiotics. This finding suggests that a significant proportion of the population perceives a lack of knowledge or understanding of the potential benefits and applications of psychobiotics in mental health. In addition, the data presented shows that a substantial majority, 64.2% (*n* =  274/427) of respondents stated that further research is needed in this area.

The results highlight that the field of psychobiotics is still emerging. Despite increased interest and first clinical indications of potential benefits, robust evidence remains limited. Continued research is needed to clarify their mechanisms of action, validate efficacy in diverse populations, and define appropriate clinical applications. The public uncertainty reflected in this study highlights not only a scientific but also a communication gap. Strategies are needed to translate complex research into accessible and reliable information to address this problem. Community‐based education, working with health professionals, and using culturally adapted public health messages can help build trust and increase engagement, particularly among populations with lower levels of formal education.

Regarding studies conducted in Romania on psychobiotics, a study at a private nutrition practice in Oradea, Romania, between 2020 and 2022 investigated the effects of psychobiotics on neuropsychiatric manifestations in children with multiple neurotransmitter disorders. The study included 135 patients aged between 5 and 18 with different gastrointestinal disorders. They received personalized treatments with probiotics, and the development of neuropsychiatric symptoms such as hyperactivity, aggression, and lack of concentration was monitored throughout the study. The study reported that psychobiotics significantly reduced levels of hyperactivity and aggression and improved concentration in children [43].

Although studies in Romania in this area are limited, numerous worldwide clinical trials have demonstrated the positive effects of psychobiotics. Recent studies have shown that psychobiotics can affect proteins and neurotransmitters like GABA (gamma‐aminobutyric acid), serotonin, glutamate, and brain‐derived neurotrophic factor (BDNF), lending credence to the idea of a biochemical and physical link between the intestines and the brain. These chemicals all significantly impact mood stabilization, cognitive function, learning, and memory, as well as the balance between inhibitory and excitatory brain signals [10, 44, 45].

However, there is still controversy about the usefulness of psychobiotics in reducing symptoms of depression. A meta‐analysis evaluating 10 clinical trials encompassing 1349 participants found no statistically significant impact of probiotic supplementation on mood compared to placebo. The study underscored several methodological limitations in the existing literature, such as inconsistencies in probiotic dosages, bacterial species, and strain combinations, which hinder meaningful comparisons across trials. Furthermore, the generalizability of these findings to individuals suffering from depression is questionable, as most randomized controlled trials have focused on healthy populations [39]. Contradicting earlier findings, recent evidence from 2024 indicates that probiotics could be effective in managing major depressive disorder, either as an adjunct to antidepressant treatment or as a standalone therapy. This conclusion emerged from a systematic review and meta‐analysis of 42 trials, suggesting a potential role for psychobiotics in the treatment of depression [46]. Additionally, a 2024 meta‐analysis focusing on strain‐specific effects (12 RCTs, *n* = 707) reported a significant reduction in depressive symptoms when assessed with the Beck Depression Inventory (BDI) (mean difference −2.69; 95% CI –4.22 to –1.16; *p* < 0.001), particularly for Lactobacillus and Bifidobacterium strains. Nevertheless, this effect was not consistently observed when other scales such as the Hamilton Depression Rating Scale (HAMD), Depression Anxiety Stress Scales (DASS), or Montgomery–Asberg Depression Rating Scale (MADRS) were used, highlighting the importance of standardized outcome measures and the need for further strain‐specific clinical research. Future research must address current gaps through high‐quality, large‐scale randomized trials that standardize intervention protocols and explore long‐term effects.

### 3.4 Incidence of Anxiety and Depression Symptoms in the Last 6 Months

According to the WHO, one of the essential components of mental health is the ability to participate in meaningful and creative activities [47]. However, the European Brain Council's Value of Treatment research reveals worrying statistics: only 52% of depressed patients receive a diagnosis, and even though 62% of those diagnosed receive treatment, only 33% have positive outcomes, and another 33% have poor outcomes. Alarmingly, only 12% of those who receive treatment consult a psychiatrist or specialist [4]. In addition, only 30% of depressed patients receive any treatment, and even fewer—only 40%—receive adequate medical care [48].

**Figure 10:**
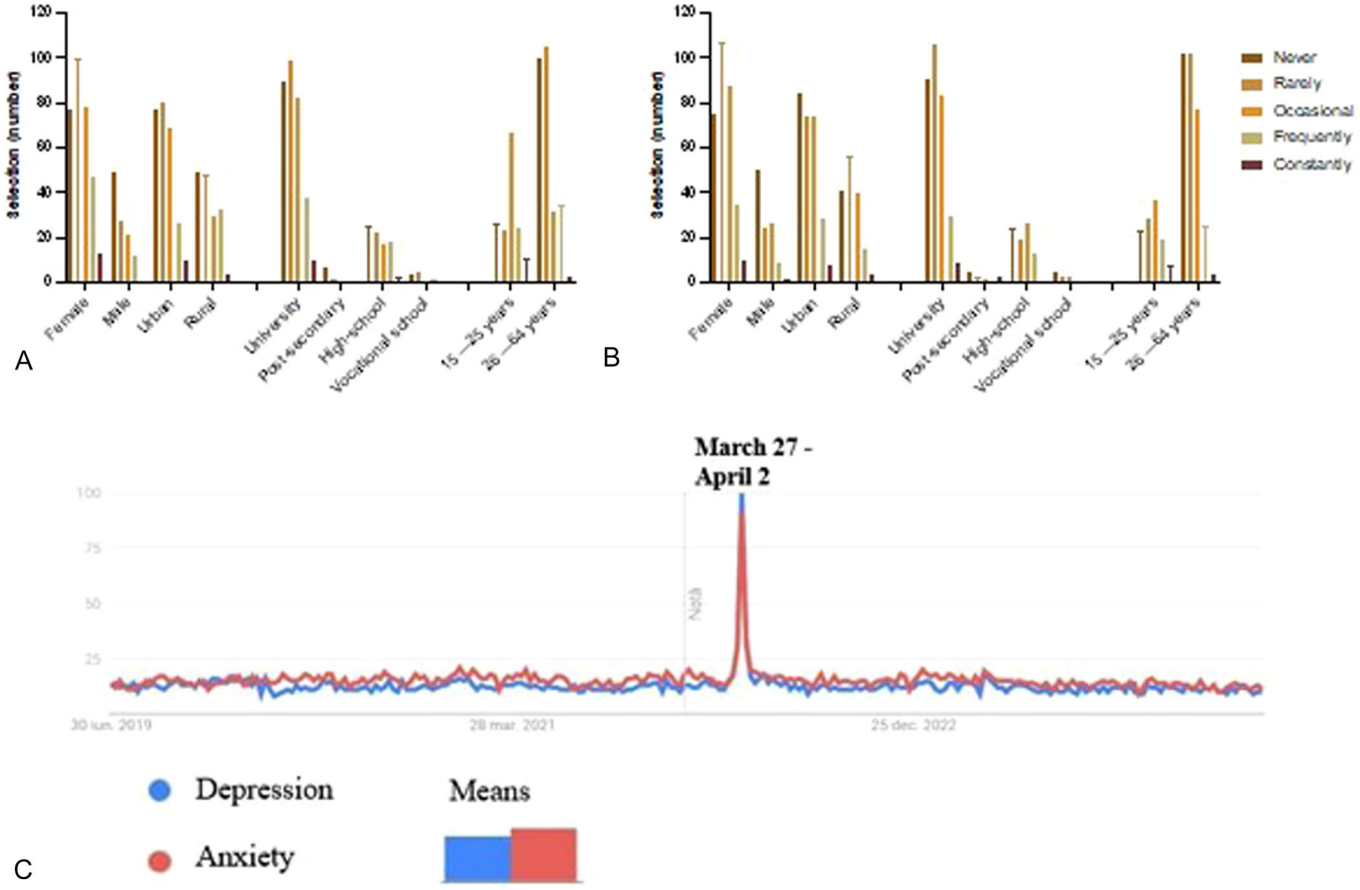
Frequency of (A) anxiety and (B) depression symptoms in the last 6 months correlated with gender, geographic origin, education level, and age; (C) Google Trend data regarding interest in terms like probiotics and psychotics. Responses were recorded using a 5‐point Likert scale: 1 = Never, 2 = Rarely, 3 = Occasionally, 4 = Frequently, 5 = Constantly.

As shown in Figure 10A,B, the survey also investigated the frequency of depression and anxiety symptoms experienced by participants over the last 6 months. The data shows that about 29.30% (*n* = 125/427) of respondents never experienced symptoms of depression, and 29.60% (*n* = 126/427) never experienced symptoms of anxiety, indicating that a substantial proportion of the population does not suffer from these conditions. Symptoms of depression were rarely experienced by 30.80% (*n* = 131/427) of respondents, while 30% (*n* = 128/427) reported rare symptoms of anxiety, suggesting a similarly low incidence for both conditions.

Occasional symptoms were reported more frequently for depression (26.80% *n *= 114/427) compared to anxiety (23.20% *n *= 99/427), indicating a slightly higher prevalence of periodic depressive symptoms. However, when it comes to more frequent manifestations, anxiety appears to be more prevalent: 13.80% (*n *= 59/427) of respondents reported experiencing frequent anxiety symptoms, compared to 10.3% (*n *= 44/427) for depression. Constant symptoms were reported by a small percentage of respondents for both conditions, with 2.80% (*n *= 12/427) for depression and 3.30% (*n *= 14/427) for anxiety. Although these percentages are relatively modest, they highlight a relevant subset of individuals who may be struggling with persistent symptoms. This trend is further supported by Google Trends data (Figure 10C), which shows sustained public interest in the terms “anxiety” and “depression” over the past 5 years in Romania. Notably, both terms peaked during the week of March 27–April 2, coinciding with the end of the COVID‐19 pandemic. This peak reflects increased public concern regarding mental health during and immediately after the pandemic period, particularly in relation to anxiety‐related symptoms.

When asked if they had ever sought or received psychological help for depression or anxiety, 79.10% (*n* = 337/427) answered in the negative, 20.20% (*n* = 86/427) answered in the affirmative, and 0.70% (*n* = 3/427) preferred not to answer. Interestingly, the percentage of those who reported frequent anxiety (10.30% *n* = 44/427) and depression (13.80% *n* = 59/427) is lower than the percentage of those who accessed counseling (20.50%, *n* = 88/427). Although they reported symptoms of anxiety or depression, 79.10% of respondents never sought psychological help. This considerable discrepancy between the presence of symptoms and the low rate of help‐seeking behavior may be attributed to the cultural stigma surrounding mental health in Romania, lack of trust in mental health services, economic constraints or limited availability of qualified professionals, especially in rural areas. These factors represent structural and social barriers that prevent people from accessing appropriate care, even when symptoms are present.

This finding suggests that some participants sought counseling even if they were not experiencing frequent anxiety or depression, possibly for preventive purposes or other mental health problems. As an alternative or addition to classical therapy, psychobiotics are promising in the field of depression [49]. They have this potential because they affect gut flora and the metabolism of neurotransmitters such as the serotonin‐producing tryptophan. Investigating the effects of *Bifidobacterium breve* CCFM1025 on patients with major depressive disorder (MDD), a study conducted by Tian et al. [17] and Xu et al. [50] found that the probiotic had a favorable effect on gut flora and tryptophan metabolism, in addition to alleviating the patient's depression. Furthermore, researchers conducted a randomized trial to determine the effectiveness of probiotic supplements for patients with MDD. The investigation focused on depressive symptoms, gut microbiota composition, and brain‐related changes. Patients using antidepressants for the first time received either a placebo or a multi‐strain probiotic for an entire month. When comparing the probiotic and placebo groups on the Hamilton depression rating scale, the former showed a considerably statistically significant reduction in depressive symptoms. The variety of gut bacteria, particularly *Lactobacillus*, was also improved by probiotics. This study highlights the potential of probiotic treatments in treating MDD [51].

Utilizing data from 13 clinical trials, the 2023 meta‐analysis by Zhang et al. [52] shed light on the overall efficacy of psychobiotics in treating MDD. Despite significant antidepressant effects that have been observed with probiotics, the effectiveness of prebiotics and synbiotics in alleviating depression remains less clear and requires further investigation. Currently, there are limited studies on synbiotics, with only one showing significant antidepressant effects. At the same time, three prebiotic trials revealed no significant differences from placebo, indicating a need for further clinical trials to assess their potential efficacy in improving depressive symptoms. Although emerging studies show promising results regarding the use of psychobiotics in managing depressive symptoms, the field remains in its early stages. The clinical evidence is still evolving, and caution should be exercised when interpreting these findings. Larger and more rigorous trials are needed to confirm efficacy, where current data are limited.

Research conducted in Australia used the Patient Health Questionnaire 9 (PHQ‐9) and General Anxiety Disorder 7 (GAD‐7) to assess mental health during COVID‐19 restrictions. It surveyed responses from 13,829 participants and provided a comprehensive breakdown of depression and anxiety symptoms across age and gender groups. The study highlighted the significant prevalence of moderate to severe symptoms among the general population, underlining the broad impact of the pandemic on mental well‐being [53].

### 3.5 Sources of Reliable Information and Factors Influencing the Decision to Take Psychobiotics

**Figure 11:**
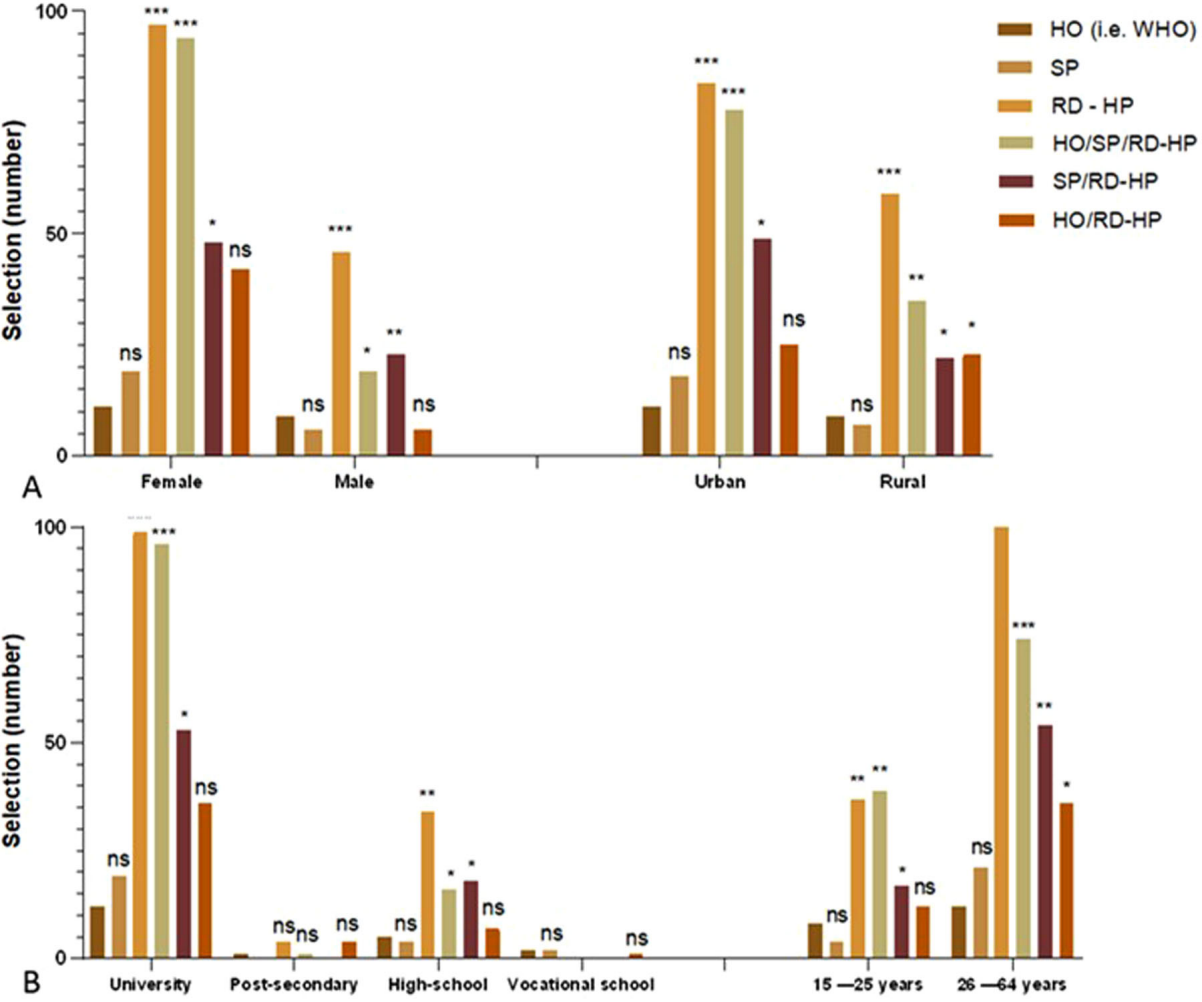
Sources of information considered the most reliable regarding the benefits associated with psychobiotic consumption (HO ‐Health Organizations like WHO; SP ‐Scientific Publications; RD‐HP Recommendations from Doctors or Health Professionals). The results are expressed as mean values ± SD (*n* = 3). Tukey's multiple comparison tests were applied, with *p* values provided when *p* > 0.05, and the following symbols are used for interpretation: ***p < 0.001, **p < 0.01, *p < 0.05; ns > 0.05. Preferred sources of information on psychobiotics, as reported by the respondents, according to socio‐demographic factors. HO = Health Organization (e.g., WHO); SP = Scientific Publications; RD–HP = Registered Dietitian or Health Professional; HO/SP/RD–HP = combination of Health Organization, Scientific Publications, and Health Professional; SP/RD–HP = Scientific Publications and Health Professional; HO/RD–HP = Health Organization and Health Professional. (A) Distribution of preferred information sources by gender and living environment. (B) Distribution of preferred information sources by education level and age group. Statistical significance: *p < 0.05; **p < 0.01; ***p < 0.001; ns, not significant.

Understanding how consumers access and evaluate information about psychobiotics is key to developing effective health communication strategies. When assessing perceived trust, demographic factors such as gender, geographic location, education, and age play a crucial role. As shown in Figure 11, scientific publications and health organizations (e.g., WHO) are considered the most trustworthy sources of information, especially by women, urban residents, individuals with higher levels of education, and older adults. In contrast, advice from medical professionals tends to have greater influence among rural populations and male respondents, reflecting trust in personalized, authoritative recommendations. Interestingly, these findings differ from those of previous studies in other regions, where informal channels such as websites, television, and social media are often the primary information sources [54, 55]. In our survey, only one respondent mentioned social media as influential, suggesting a notable level of doubt about informal platforms in Romania. This may reflect broader socio‐cultural patterns in which Romanian consumers, particularly those with higher education, tend to prefer formal and validated health information.

Medical advice and scientific evidence were identified as the two most influential factors in the decision to take psychobiotics. Specifically, 315 participants cited medical professionals' advice as their primary influence, while 263 emphasized scientific validation. These figures suggest that evidence‐based recommendations have a significant impact on consumer decisions and are preferred over commercial or informal advice that lacks scientific support. In contrast, social networks and consumer experiences are virtually dismissed as reliable information sources, with only one respondent each citing these as influential. This starkly contrasts with the widespread use of social media, suggesting a skepticism or lack of trust in informal online platforms for making health‐related decisions. Personal stories and user reviews also failed to significantly impact decision‐making, reinforcing the preference for more formal, evidence‐based sources. Interestingly, according to another study, the significant sources of information for Indian college students were television commercials and social media; however, with a few exceptions, the term “prebiotics” was unfamiliar to the same class of students [56].

Similarly, price and brand reputation played a limited role in consumer decisions regarding psychobiotics. Only 80 respondents cited price as a factor, and just 23 referenced brand reputation. This relatively low influence should be understood in the context of broader Romanian health consumer behavior, which tends to prioritize trust and scientific validation over affordability or branding. Meanwhile, manufacturer health claims, particularly those aligned with EU regulatory standards, carried significant weight. The EU requirement for health claims to be supported by scientific evidence greatly enhances consumer confidence and informed decision‐making [19].

The reporting of this study is aligned with the STROBE recommendations, which support clarity and completeness in observational research. These findings highlight a clearly defined hierarchy of information sources. Romanian consumers place the most trust in professional advice and empirical evidence, with the least trust in informal, commercial, or peer‐oriented sources. This information can be leveraged in future public health strategies, which should emphasize credible and regulated communication channels and avoid over‐reliance on social media or promotional content when addressing the issue of psychobiotics.

## 4 Limitations and Challenges

To the best of our knowledge, it is the first investigation in Romania to assess public opinion, knowledge, and acceptability of psychobiotics. Although the findings provide valuable preliminary insights, some methodological limitations must be acknowledged. First, the exclusive use of an online survey may have excluded individuals with limited digital access or low computer literacy, particularly among older or rural populations. Second, self‐reported data inherently carry the risk of social desirability bias and reduced accuracy. Participants may unintentionally or intentionally provide responses that reflect perceived expectations rather than genuine beliefs or experiences. Although attention filters and logical consistency checks were embedded in the survey design to improve data quality, a risk of rushed or inattentive responses remains. The reliability of the data thus depends largely on participant honesty, comprehension, and engagement.

Additionally, the study did not employ multivariate statistical analysis, which limits the ability to assess the independent effects of demographic variables. Future studies should benefit from the use of multivariate models to better understand the complex interactions between these variables and to strengthen the interpretative depth of the findings.

To approach these issues in future studies, the inclusion of individual, semi‐structured interviews is strongly recommended. In contrast to online surveys, qualitative interviews favor direct interaction between researchers and participants, which facilitates trust and allows for probing of participants' reasoning and knowledge gaps. Interviews can also allow for the clarification of unclear questions in real‐time. They can be tailored to individual levels of understanding, making them particularly valuable when addressing less familiar or complex concepts, such as psychobiotics. Addressing these limitations through a mixed‐methods approach and more robust statistical modeling will be essential for advancing research in this field. Such improvements can help refine public health strategies, strengthen scientific understanding, and support inclusive, evidence‐based communication efforts around psychobiotics.

## 5 Conclusions

This study found that Romanian consumers generally demonstrated low familiarity with psychobiotics. Although awareness of probiotics and their general health benefits was high, detailed knowledge regarding the specific role of psychobiotics in mental health, particularly in managing anxiety and depression, remained limited. Nevertheless, there was notable public interest in the mental health potential of psychobiotics, and respondents expressed favorable attitudes toward their use.

The findings revealed a strong preference for evidence‐based information and professional validation. Scientific evidence and recommendations from healthcare providers significantly influenced participants' decision‐making, whereas informal sources such as social media or personal reviews played a negligible role. Factors such as price and brand were considered secondary, with consumers prioritizing credibility and trustworthiness.

A significant proportion of respondents expressed a need for more accessible, reliable information and greater product availability. This highlights the importance of coordinated educational and communication efforts. Public health authorities should consider launching targeted campaigns to raise awareness of the gut–brain axis and the potential role of psychobiotics. Similarly, health education programs, particularly in schools and universities, could incorporate content on the gut microbiota and its relationship to mental health. In clinical settings, healthcare professionals and pharmacists should be equipped with up‐to‐date, evidence‐based resources to guide consumers and patients interested in these products.

Improving access to reliable information and implementing educational strategies through trusted institutional channels will help foster informed consumer choices. These actions may support the responsible integration of psychobiotics into broader preventive and therapeutic mental health initiatives.

## Author Contributions

Cocean Ana‐Maria and Dan Cristian Vodnar conceptualized the study and developed the questionnaire. Bernadette‐Emoke Teleky and Cocean Ana‐Maria conducted the survey and handled data interpretation. Cocean Ana‐Maria compiled the results and literature and drafted the manuscript. All authors reviewed and approved the final version.

## Ethics Statement

The study followed the ethical principles of the Declaration of Helsinki and national legislation (Law 206/2004). Ethical approval was waived by the Bioethics Committee of the University of Agricultural Sciences and Veterinary Medicine of Cluj‐Napoca, as the research did not involve any clinical intervention or the collection of identifiable personal data.

## Consent

All participants provided informed consent before completing the anonymous online questionnaire.

## Conflicts of Interest

The authors declare no conflicts of interest.

## Transparency Statement

The corresponding author, Dan Cristian Vodnar, affirms that this manuscript is an honest, accurate, and transparent account of the study being reported; that no important aspects of the study have been omitted; and that any discrepancies from the study as planned (and, if relevant, registered) have been explained.

## Data Availability

The data supporting the findings of this study can be obtained from the corresponding author upon reasonable request. All authors have read and approved the final version of the manuscript Dan Cristian Vodnar had full access to all of the data in this study and takes complete responsibility for the integrity of the data and the accuracy of the data analysis.
